# The Preparation of De-Hæmoglobinised Blood Films

**Published:** 1903-02-21

**Authors:** 


### THE PREPARATION OF DE-HÆMOGLOBINISED BLOOD FILMS.

THE microscopic examination of blood has now been conclusively shown to be essential to the diagnosis of many serious affections, and of conspicuous service in affording indications for prognosis. It is therefore particularly desirable that means should be found whereby methods may be simplified and rendered as convenient as possible for speedy and accurate clinical work. Major Ross's recently introduced method of preparing de-hæmoglobinised blood films is certainly a step in the right direction.[1] It is not only of great assistance in facilitating the search for malarial organisms, but as Dr. Lovell Gulland has recently shown [2] is of much assistance in making a differential count in cases accompanied by leucocytosis. Professor Ross's method is as follows:—A large drop of blood, amounting to, say, 20 cubic millimetres is taken up on the glass slide or cover glass and is slightly spread out by means of the needle or lancet. It is then allowed to dry, either naturally or over a flame (without heating so much as to fix the hæmoglobin). The result is a thick and dried film in which about 20 cubic millimetres of blood (instead of the one cubic millimetre generally used) are disposed over an area which is not larger than that usually examined by the ordinary processes. As soon as the film is perfectly dry, by means of a glass rod add sufficient aqueous solution of eosin to cover the film. The solution is the one which is usually employed in order to obtain the Romanowsky effect. It is allowed to remain for about a quarter of an hour. This period should be inversely proportionate to the strength of the solution. As the film has not been fixed the solution of eosin will take out the hæmoglobin of the dried corpuscles, and, at the same time, will stain the residual mass consisting of the stomata of the corpuscles, the leucocytes, blood plates, and parasites. The stain is then washed off by a very gentle stream of water. As the film has not yet been fixed to the glass it is most essential that no strong current of water should be employed else the whole residual mass will be detached. As soon as the eosin solution has been washed out a weak solution of the methylene blue which is employed for the Romanowsky stain is allowed to run over the film. The blue is permitted to remain only for a few seconds, the time varying inversely with the strength of the solution. Especial care is necessary not to stain the preparation too darkly. The blue must be washed off very carefully. The stained film is then dried and mounted in Canada balsam, and the preparation is complete.

[1] Lancet, Jan. 10, 1903.
[2] Scottish Medical and Surgical Journal, February 1903.

